# Supplementary material for: Causal Relationship between Aging and Anorexia Nervosa: A White-Matter-Microstructure-Mediated Mendelian Randomization Analysis
Source: Biomedicines. 2024 Aug 16;12(8):1874. doi: 10.3390/biomedicines12081874 (PMC11351342; doi:10.3390/biomedicines12081874)
Supplement: Supplementary file 1 [file biomedicines-12-01874-s001.zip › Supplementary Figure/Figure S8/Leaveoneout plot of MR analysis for TL to ubm-b-1766.pdf]

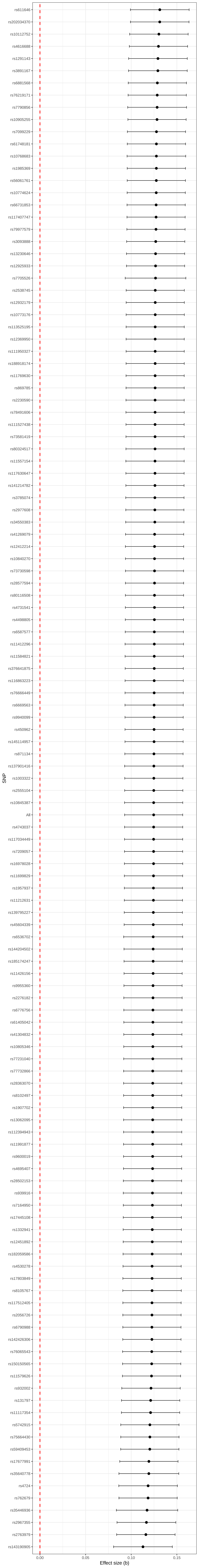
rs611646
rs202034370
rs10112752
rs4616688
rs1291143
rs3891167
rs6881568
rs76219171
rs7790856
rs10905255
rs7099229
rs61748181
rs10768683
rs1985369
rs56061761
rs10774624
rs66731853
rs117407747
rs79977579
rs3093888
rs13230646
rs12925933
rs7705526
rs2538745
rs12932179
rs10773176
rs113525195
rs12369950
rs111950327
rs188918174
rs11769630
rs869785
rs2230590
rs78491606
rs111527438
rs73581419
rs80324517
rs11557154
rs117630647
rs141214782
rs3785074
rs2977608
rs34550383
rs41269079
rs12412214
rs10840270
rs73730598
rs28577594
rs80116508
rs4731541
rs4498805
rs6587577
rs11412296
rs11584821
rs376641875
rs116863223
rs76666449
rs6669563
rs9940099
rs450962
rs145114957
rs871134
rs137901416
rs1003322
rs2555104
rs10845387
A8
rs4743037
rs117034449
rs7209057
rs16978028
rs11699829
rs1957937
rs11212631
rs139795227
rs45604339
rs6536702
rs144204502
rs185174247
rs11426156
rs9955360
rs2276182
rs6776756
rs61405042
rs41304832
rs10805346
rs77231040
rs77732866
rs28363070
rs8102497
rs1907702
rs13062095
rs112394943
rs11991877
rs9600019
rs4695407
rs28502153
rs939916
rs7164950
rs17445108
rs1332941
rs12451892
rs182059586
rs4530278
rs17803849
rs8105767
rs117512405
rs2056726
rs6790988
rs142426306
rs76065543
rs150150565
rs11579626
rs932002
rs131797
rs111117354
rs5742915
rs75664430
rs59409453
rs17677991
rs35640778
rs4724
rs762679
rs35446936
rs2967355
rs2763979
rs143190905
SNP
0.00
0.05
0.10
0.15
Effect size (b)
